# Comparative transcriptome analysis of scaled and scaleless skins in *Gymnocypris eckloni* provides insights into the molecular mechanism of scale degeneration

**DOI:** 10.1186/s12864-020-07247-w

**Published:** 2020-11-27

**Authors:** Xiu Feng, Yintao Jia, Ren Zhu, Kemao Li, Zhongzhi Guan, Yifeng Chen

**Affiliations:** 1 Institute of Hydrobiology, Chinese Academy of Sciences, Wuhan, 430072 China; 2 QingHai Provincial Fishery Environmental Monitoring Center, Xining, 810012 China

**Keywords:** *Gymnocypris eckloni*, Transcriptome, Skin, Scale degeneration

## Abstract

**Background:**

The scale degeneration is thought to be related to the adaptation to the extreme environment with cold climate and high-altitude in schizothoracine fishes. *Gymnocypris eckloni*, a schizothoracine fish living in plateau waters with the elevation above 2500 m, is nearly esquamate and only covered with shoulder scales and anal scales, making it a good model species to study the molecular mechanism of scale degeneration.

**Results:**

The transcriptomes of shoulder scaled skins (SSS), anal scaled skins (ASS) and scaleless skins (NSS) were sequenced and analyzed in *G. eckloni* at the age of 1 year. Histological examination showed that shoulder scale had completed its differentiation and anal scale just initiated the differentiation. A total of 578,046 unigenes were obtained from the transcriptomes, with 407,799 unigenes annotated in public databases. A total of 428 and 142 differentially expressed unigenes (DEUs) were identified between SSS and NSS, and between ASS and NSS, respectively, with 45 DEUs that were overlapped. Annotation analysis indicated that these DEUs were mainly enriched in Gene Ontology (GO) terms and KEGG pathways associated with bone and muscle formation, such as myofibril, contractile fiber, cytoskeletal protein binding, muscle structure development, cardiac muscle contraction, hypertrophic cardiomyopathy (HCM) and calcium signaling pathway.

**Conclusions:**

Our results would provide insights into the molecular mechanisms of scale degeneration in *G. eckloni* and other congeneric fishes. In addition, the transcriptome data provides candidate genes and markers for future studies.

## Background

The scales in fish species refer to the dermis-derived structures located within the skin, and are classified into placoid, ganoid and elasmoid (cycloid and ctenoid) [1–3]. Most scales in teleosts belong to the elasmoid type, which is a highly derived type of scale [1]. Many studies have described the scale formation process at the tissue and cellular level [1, 4–7], but little is known at the molecular level. Only several candidate genes are supposed to contribute the fish scale formation. In zebrafish (*Danio rerio*), whole-mount in situ hybridization revealed that *sonic hedgehog* (*shh*) may be involved in the control of scale morphogenesis and differentiation [1]. In medaka (*Oryzias latipes*), a mutation at the *rs-3* locus encoding *ectodysplasin-A receptor* (*EDAR*) leads to almost complete loss of scales, indicating that EDAR is required for scale development [8, 9]. Based on phylogenetic analyses, *ectodysplasin-A* (*Eda*) and *secretory calcium-binding phosphoproteins* (*SCCPs*) are also supposed to be associated with the scale development in fish species [10–12]. However, very few studies have been conducted using genomic or transcriptomic analyses.

The transcriptome represents the set of all transcripts expressed in one cell or a population of cells. With the development and popularization of the next-generation sequencing technologies (NGS), whole transcriptome sequencing or RNA sequencing (RNA-seq) has been widely used for transcriptome analysis at massive scale [13]. Comparative transcriptome analysis provides a powerful tool for dissecting the relationship between genotype and phenotype, increasing our understanding of the molecular mechanisms involved in physiological process and environmental adaptation [14]. For example, comparative transcriptome analysis by RNA-seq has identified genes and pathways associated with growth [15], gonad development [16], immune response [17] and skin color differentiation [18] in fish species.

The schizothoracine fishes (Teleostei: Cyprinidae) are mainly distributed in the Qinghai Tibetan Plateau (QTP) and its surrounding areas, and are the largest group of the QTP ichthyofauna [19]. Among them, *Gymnocypris* fishes mainly live in the cold and high altitude area on the QTP, which are nearly esquamate, only covered with 3–4 rows of shoulder scale, and two lines of anal scale [19, 20]. The degenerated scale and incrassated skin are two characteristics of *Gymnocypris* fishes which related to the adaptation to the extreme environment with cold climate and high-altitude. Uncovering the molecular basis that controlling the scale degeneration would provide new insights into how the *Gymnocypris* fishes adapt to the extreme environment of the QTP. The presence of shoulder scales and anal scales indicate that the scale degeneration may be involved in gene expression regulation rather than the loss of a single or a few genes. Previous studies of *Gymnocypris* fishes have made great progress in phylogenetics, biogeography and ecology [21–24]. However, the molecular mechanism of scale degeneration is still not studied.

*Gymnocypris eckloni*, a representative species belonging to the genus *Gymnocypris*, is a native fish species in the upper reaches of the Yellow River with the elevation above 2500 m. In this study, we sequenced and analyzed the transcriptomes of scaled and scaleless skin tissues in *G. eckloni*. The aim of this study is de novo assembly of the transcriptome and identification of differentially expressed unigenes that may be involved in scale degeneration in *G. eckloni*.

## Results

### Histology observation of skin tissues

**Fig. 1:**
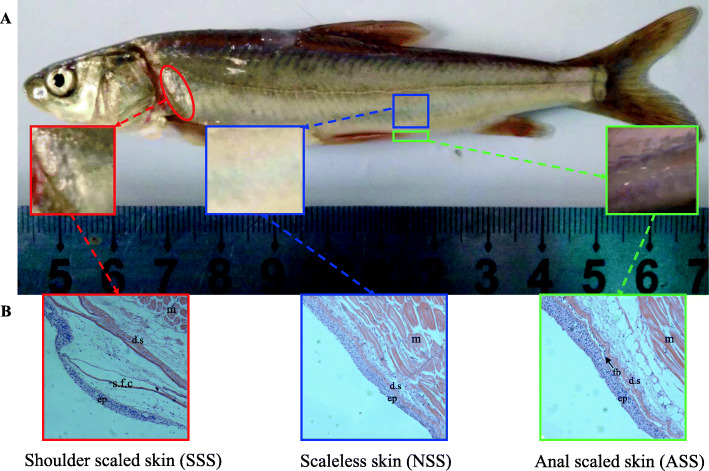
The diagrammatic drawing of sample positions (**a**) and histology observation (magnification × 200) (**b**) of skin tissues in *G. eckloni*. ep, epidermis; d.s, dermal stroma; m, muscle; s.f.c, scale-forming cells; fb: fibroblast

At age of 1 year, juvenile specimens of *G. eckloni* (with the average body weight and body length of 15.01 g and 10.23 cm) showed obvious scales on the shoulder skin but only slight folds on the anal skin (Fig. 1). Histological examination showed that the shoulder scale had completed its differentiation, and its posterior region had protruded into the epidermis which formed a fold. The anal scale had just reached at the early stage of development, the scale papilla had differentiated in the dermis. The scaleless skin showed no morphogenesis of scale development.

### Transcriptome sequencing, assembly and annotation

The transcriptomes of shoulder scaled skins (SSS), anal scaled skins (ASS) and scaleless skins (NSS) from three fish individuals were sequenced by using Illumina paired-end sequencing technology. In total, 505.99 million raw reads were generated from all nine tissue samples (Table 1). After trimming and filtering, a total of 493.47 million clean reads were obtained, with the average number of reads of 54.83 million for each tissue sample (Additional file 1). A total of 771,455 transcripts from 433,844 ‘genes’ were de novo assembled by Trinity software [25]. After clustering by CD-HIT-EST [26], 578,046 unigenes were obtained with the average length and N50 length of 747 bp and 1193 bp.

**Table 1:** Summary for the transcriptome of *G. eckloni* using Illumina RNA-seq

Prameters	Data
Number of raw reads	505,993,248
Number of clean reads	493,470,036
Number of unigenes	578,046
Mean length of unigenes (bp)	747
N50 length of unigenes (bp)	1193

**Fig. 2:**
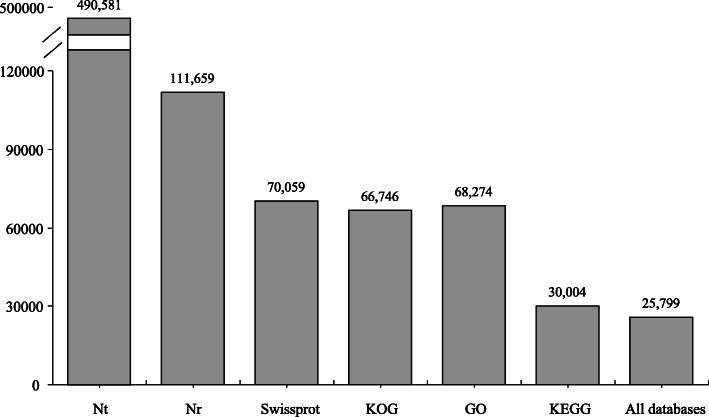
The number of unigenes annotated with Nt, Nr, Swissprot, KOG, GO and KEGG databases

The unigenes were annotated based on the public databases. A total of 490,581, 111,659, 70,059, 66,746, 68,274 and 30,004 unigenes were assigned to NCBI non-redundant nucleotide sequences (Nt), non-redundant protein sequences (Nr), Swissprot, Cluster of Orthologous Groups of proteins (KOG), Gene Ontology (GO) and KEGG Ortholog database (KEGG), respectively, with 25,799 unigenes annotated in all databases and 407,799 unigenes annotated in at least one database (Fig. 2). GO annotations generated 66 level 2 GO terms under three functional categories: cellular component, molecular function and biological process (Additional file 2). For KEGG annotation, the ‘signal transduction’ pathway had the largest number of unigenes (*n* = 2672), followed by ‘global and overview maps’ (*n* = 2121) and ‘infectious disease: viral’ (*n* = 1422) (Additional file 3). Similar results of GO and KEGG annotations have also been reported in a previous study [27].

### Identification of differentially expressed unigenes (DEUs)

**Fig. 3:**
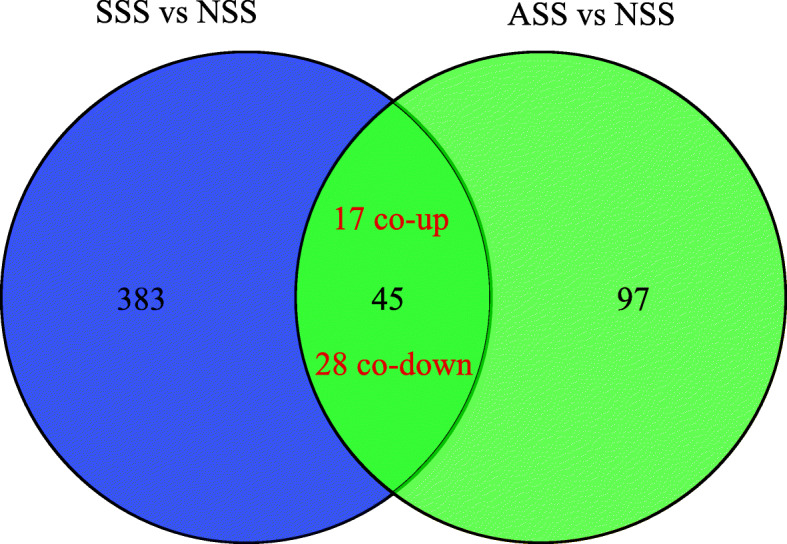
The venn diagram of differentially expressed unigenes (DEUs). SSS, shoulder scaled skins; ASS, anal scaled skins; NSS, scaleless skins

**Fig. 4:**
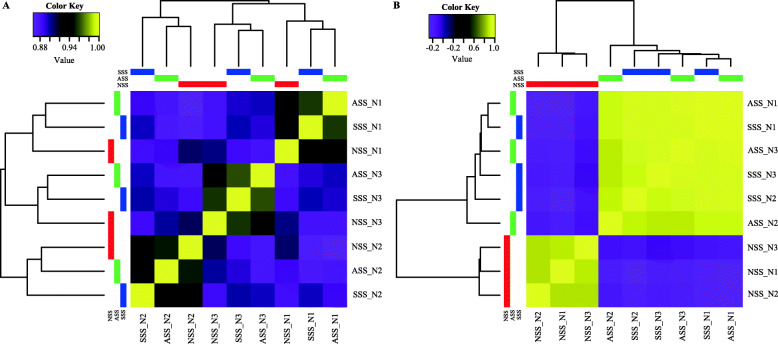
Heat map showing the pairwise Spearman correlations among various tissues based on expression profiles of all unigenes (**a**) and DEUs (**b**)

Based on the criteria of |log_2_FC| ≥ 2 and *p*-value ≤0.001, a total of 428 and 142 DEUs were identified between SSS and NSS, and between ASS and NSS, respectively. Compared with NSS, SSS had 75 up-regulated and 353 down-regulated unigenes, and ASS had 39 up-regulated and 103 down-regulated unigenes. The Venn diagram showed that 17 and 28 unigenes were up-regulated and down-regulated, respectively, in both SSS and ASS compared with NSS (Fig. 3). Based on the global expression profiles, the samples were clustered into three groups corresponding to the three fish individuals (Fig. 4a). However, based on the expressions of DEUs, the samples were clustered into two groups representing scaleless and scaled skins (Fig. 4b).

### Functional annotation of DEUs

A total of 384, 349 and 207 DEUs for SSS, and 127, 110 and 58 DEUs for ASS were assigned to Nr, GO and KEGG databases, respectively (Additional file 4). For overlapped DEUs, 38, 33 and 19 unigenes were annotated to the three databases.

GO enrichment analysis showed that DEUs for SSS were mainly enriched in ‘myofibril’ (GO:0030016), ‘contractile fiber’ (GO:0043292) and ‘actin cytoskeleton’ (GO:0015629) under cellular component, ‘actin binding’ (GO:0003779), ‘actin filament binding’ (GO:0051015) and ‘cytoskeletal protein binding’ (GO:0008092) under molecular function, and ‘muscle structure development’ (GO:0061061), ‘muscle cell development’ (GO:0055002) and ‘myofibril assembly’ (GO:0030239) under biological process (Table 2). DEUs for ASS had similar enriched terms under cellular component, with much lower adjusted *p*-values compared with DEUs for SSS.

**Table 2:** GO enrichment analysis of DEUs

	DEUs between SSS and NSS			DEUs between ASS and NSS		
	GO term	N ^1^	FDR	GO term	N ^1^	FDR
CC ^2^	myofibril	96	1.2E-80	extracellular region	36	1.0E-04
	contractile fiber	96	7.1E-80	myofibril	11	7.2E-04
	sarcomere	88	2.0E-76	contractile fiber	11	7.2E-04
	contractile fiber part	89	2.2E-75	sarcomere	10	7.2E-04
	Z disc	56	1.8E-44	contractile fiber part	10	9.6E-04
	striated muscle thin filament	32	3.4E-37	interstitial matrix	3	5.7E-03
	myofilament	32	1.0E-35	melanosome membrane	3	5.9E-03
	actin cytoskeleton	74	1.1E-33	cardiac Troponin complex	2	5.9E-03
	cardiac myofibril	11	4.2E-16	Z disc	7	6.5E-03
	sarcoplasm	24	1.0E-16	cardiac myofibril	2	2.6E-02
MF ^3^	actin binding	75	1.2E-31	luteinizing hormone receptor activity	2	5.1E-03
	actin filament binding	51	1.1E-27	melanocortin receptor activity	2	1.5E-02
	cytoskeletal protein binding	93	2.3E-27	sulfur compound binding	7	3.2E-02
	troponin C binding	13	5.2E-18	actin binding	12	3.3E-02
	structural constituent of muscle	20	1.9E-16	frizzled binding	3	4.6E-02
	alpha-actinin binding	18	5.4E-15	protein-hormone receptor activity	2	4.6E-02
	muscle alpha-actinin binding	15	8.9E-15	cation:cation antiporter activity	3	4.6E-02
	actinin binding	18	1.1E-13	solute:cation antiporter activity	3	4.9E-02
	FATZ binding	9	3.0E-13	potassium:proton antiporter activity	2	4.9E-02
	telethonin binding	11	1.2E-12	mono-olein transacylation activity	1	4.9E-02
BP ^4^	muscle structure development	87	3.3E-59	muscle structure development	12	6.0E-03
	striated muscle cell development	64	2.9E-57	luteinizing hormone signaling pathway	2	6.0E-03
	muscle cell development	65	5.2E-57	cellular response to luteinizing hormone stimulus	2	6.0E-03
	muscle system process	66	4.8E-55	tissue development	22	7.2E-03
	striated muscle cell differentiation	65	2.4E-52	response to luteinizing hormone	2	9.0E-03
	muscle cell differentiation	69	1.0E-51	sodium ion import	3	9.3E-03
	muscle organ development	65	2.3E-51	sodium ion import across plasma membrane	3	9.3E-03
	myofibril assembly	49	1.4E-50	sodium ion import into cell	3	9.3E-03
	muscle contraction	57	9.5E-48	appendage development	6	1.5E-02
	muscle fiber development	39	4.3E-37	hair follicle morphogenesis	2	2.0E-02

**Fig. 5:**
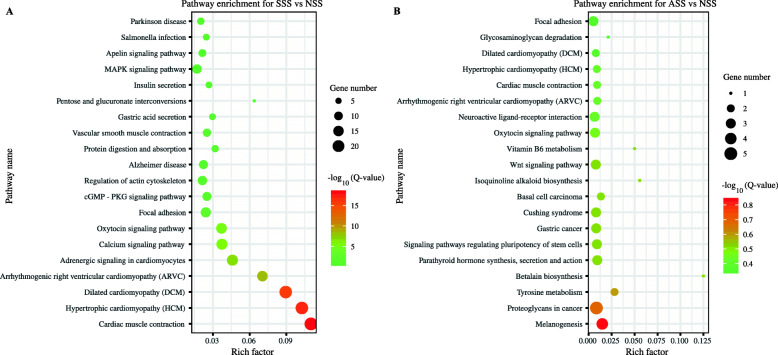
KEGG enrichment analysis of DEUs between SSS and NSS (**a**) and between ASS and NSS (**b**). SSS, shoulder scaled skins; ASS, anal scaled skins; NSS, scaleless skins

KEGG enrichment analysis showed that DEUs for SSS were mainly enriched the pathways ‘cardiac muscle contraction’, ‘hypertrophic cardiomyopathy (HCM)’, ‘dilated cardiomyopathy’ and ‘calcium signaling pathway’ (Fig. 5a). Among the top 20 of enriched pathways, six were enriched for both DEUs for SSS and DEUs for ASS (Fig. 5b). However, DEUs for SSS had much higher enrichment scores than DEUs for ASS.

The overlapped DEUs only contain 45 unigenes (Table 3). The top five up-regulated unigenes (according to log_2_FC) were UPF0575 protein C19orf67 homolog, CUB and zona pellucida-like domain-containing protein 1, sodium/calcium exchanger 1, osteocalcin and galectin-4. The top five down-regulated unigenes include melanotransferrin, tensin, myozenin-2, E3 ubiquitin-protein ligase TRIM21 and troponin I, slow skeletal muscle.

**Table 3:** The overlapped DEUs between SSS vs NSS and ASS vs NSS

Unigenes	Log_2_FC (SSS vs NSS)	log_2_FC (ASS vs NSS)	Description
DN2099_c0_g1_i7	−11.30	−11.21	Melanotransferrin
DN16257_c0_g1_i4	−10.16	−10.07	Tensin
DN10934_c0_g1_i2	−9.80	−9.70	Myozenin-2
DN5729_c1_g1_i2	−9.78	−9.60	E3 ubiquitin-protein ligase TRIM21
DN6176_c0_g2_i4	−9.75	−6.40	Troponin I, slow skeletal muscle
DN6176_c0_g3_i1	−9.67	− 9.57	Troponin I, cardiac muscle
DN10193_c0_g2_i1	− 9.42	− 9.33	Filamin-C
DN3099_c0_g1_i1	−9.26	−9.16	Myosin-16
DN6176_c0_g1_i16	−9.00	−8.91	Sodium/hydrogen exchanger 7
DN20169_c0_g1_i10	−8.98	−8.89	Ethanolamine-phosphate phospho-lyase
DN13779_c0_g1_i7	−8.37	−8.27	Voltage-dependent calcium channel subunit alpha-2/delta-2
DN25484_c0_g1_i7	− 8.23	− 8.13	Integrin alpha-7
DN5729_c1_g1_i11	−7.81	−9.58	E3 ubiquitin-protein ligase TRIM39-like
DN20918_c1_g1_i10	−7.29	−7.13	Myosin-7
DN2286_c0_g1_i5	−7.21	−9.80	LIM domain-binding protein 3
DN15718_c1_g1_i7	−6.58	−6.41	Triadin-like isoform X4
DN11289_c0_g2_i4	−5.69	−8.28	Neurofilament heavy polypeptide
DN2232_c0_g1_i16	−4.50	−6.35	Elongation factor 2
DN45284_c0_g1_i3	−4.36	−4.05	Growth/differentiation factor 8
DN5543_c0_g1_i16	−2.20	−3.38	Fibrinogen-like protein 1
DN19919_c0_g1_i2	2.50	2.55	Sclerostin
DN15430_c0_g1_i3	2.87	2.47	Homeobox protein Dlx5a
DN2890_c0_g1_i11	3.03	2.87	Pannexin-3
DN4077_c0_g1_i9	3.51	2.82	DNA-binding protein SATB2
DN78_c0_g4_i1	3.61	2.40	Lysyl oxidase homolog 4
DN8724_c0_g1_i1	3.87	3.23	Hormonally up-regulated neu tumor-associated kinase homolog A
DN1195_c0_g2_i1	3.94	2.51	Collagen alpha-2(V) chain-like
DN5111_c0_g1_i8	4.23	2.64	Collagen alpha-1(V) chain
DN13869_c0_g1_i1	5.69	5.01	Mucin-5 AC
DN10512_c0_g1_i6	6.87	7.48	Transient receptor potential cation channel, subfamily M
DN8192_c0_g1_i1	7.29	7.78	Galectin-4
DN142241_c0_g1_i6	7.81	5.62	Osteocalcin
DN12086_c0_g1_i2	8.85	8.17	Sodium/calcium exchanger 1
DN8352_c0_g1_i3	9.07	6.91	CUB and zona pellucida-like domain-containing protein 1
DN34990_c0_g1_i10	11.26	11.71	UPF0575 protein C19orf67 homolog

### Verification of gene expression

**Fig. 6:**
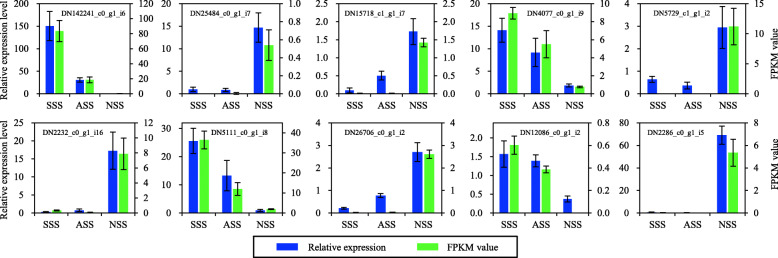
Validation of the RNA-seq expression profile by real-time reverse transcription quantitative PCR. Data were shown as mean ± SE (*n* = 3). SSS, shoulder scaled skins; ASS, anal scaled skins; NSS, scaleless skins

To assess the reliability of RNA-seq quantification analysis, ten DEUs were randomly selected for analysis using real-time reverse transcription quantitative PCR (qRT-PCR). The β-actin (DN589_c0_g1_i2) was used for the normalization in qRT-PCR, as its expression levels were confirmed to be consistent among the three groups based on RNA-seq data. The results showed that the expressions of the selected DEUs were all validated by qRT-PCR (Fig. 6). The Spearman correlation coefficient between the relative expression levels obtained by qRT-PCR and FPKM values and was 0.92 (*p* < 0.01).

## Discussion

Fish scales are derived from the dermis located within the skin [1]. The positions of the first appearance of scales vary among different fish species. In this study, the scales on the shoulder and anal of *G. eckloni* were observed, and the transcriptomes of their derived skins were sequenced and analyzed. The scales appeared first on the shoulder, which was similar to other cyprinid fishes but different from cichlid and catostomid fishes. The first scale generally appears in the anterior region of the body in cyprinid fishes [5, 28], and in the posterior region of the body in cichlid and catostomid fishes [29, 30]. The difference of scale development process may be related to species evolution and environmental adaptation.

Very large number of unigenes (578,046) was obtained from the assembled transcriptome in this study, which is similar to that previously reported in the same species (551,430) [27] as well as in another congeneric species (532,241) [24]. The main reason for this phenomenon may be that *Gymnocypris* fishes are tetraploid with large numbers of chromosomes and big genome sizes [31, 32]. Based on global expression profiles, tissue samples were clustered into groups representing fish individuals and not the skin types. An explanation may be that the scales are derived from skins, and the difference in expression between scaled and scaleless skins was smaller than that between individuals. At age of 1 year, shoulder scale completed its differentiation and anal scale just initiated the differentiation. More DEUs were detected between SSS and NSS (428) than between ASS and NSS (142), indicating that more and more genes may be involved in the process of scale development.

DEUs between scaled and scaleless skins were mainly enriched in GO terms associated with bone and muscle formation, such as ‘myofibril’, ‘contractile fiber’, ‘cytoskeletal protein binding’, and ‘muscle structure development’. These GO terms have also been reported in other studies with their functions on the development and differentiation in skin, bone and muscle [33–37]. One of the most significant up-regulated unigenes, *Osteocalcin*, is a marker of mature osteoblasts in mammals, which may be an important gene for scale formation [38, 39]. Osteocalcin is the most abundant noncollagenous bone protein of many fishes, such as common carp, grass carp and tilapia, but except for some scaleless fishes, such as channel catfish [40]. The fish scale is a type of dermal skeleton, so the genes related to scale formation in this study also are involved in bone formation [41]. The dermal bone of fish scales has been used as a model for bone research [42].

Previous studies have indicated that several genes may be involved in fish scale formation, such as *ectodysplasin-A receptor* (*EDAR*) [9], *ectodysplasin-A* (*Eda*) [12] and s*ecretory calcium-binding phosphoproteins* (*SCPPs*) [10]. In this study, these genes were all present and expressed in both scaled and scaleless skins of *G. eckloni* with no significant difference. Similarly in common carp, both *Eda* and *EDAR* were not differently expressed during scale regeneration [10]. Furthermore, the two genes were also both present and expressed in the skin of channel catfish, a scaleless fish [10]. These findings indicate that the expression of *Eda* and *EDAR* is present in both the scaled skin and the scaleless skin, and is not the only requirement for scale development in fish species. *G. eckloni*, a fish species belonging to Cyprinidae, is not entirely scaleless, and is covered with shoulder scales and anal scales. The scale degeneration in this fish are not caused by the loss of key genes reported previously, and may be related to the expression regulation of genes identified in this study. Such regulation mechanism was developed to adapt to the extreme environment of the QTP, and may be mediated by transcriptional or post-transcriptional factors, such as methylation, transcription factors (TF) and microRNAs (miRNAs).

## Conclusions

In summary, the transcriptomes of scaled and scaleless skins were firstly sequenced and compared in *G. eckloni*. The reference transcriptome with 578,046 unigenes was de novo assembled. A number of differentially expressed unigenes were identified between scaled and scaleless skins. These unigenes were mainly involved in GO terms and KEGG pathways associated with bone and muscle formation. Our results would provide insights into the molecular mechanisms of scale degeneration in *G. eckloni* and other congeneric fishes.

## Methods

### Fish materials

The parent population of *G. eckloni* used for artificial propagation and releasing was raised at the Suzhi station of fish propagation (Haidong, China). A progeny population were generated by artificial propagation in May 2018. The progenies was then raised in a 0.3 ha muddy pond and fed three times daily at QingHai Provincial Fishery Environmental Monitoring Center (Xining, China). After 1 year of culture, the neonatal shoulder scales and anal scales could be observed by optical microscope. Three fish individuals were collected after they were euthanized with an overdose of MS222 (100 mg/L). Skin tissues of shoulder scales, anal scales and no scales were sampled from each individual. One part of them was fixed in 4% paraformaldehyde, sectioned (6 mm) and stained by standard hematoxylin-eosin (H&E) staining to examine the stages of scale development. Another part was immediately frozen in liquid nitrogen.

### RNA extraction, library construction and sequencing

Total RNA was extracted using Trizol reagent (Invitrogen, Carlsbad, CA, USA) according to the manufacturer’s instructions. RNA degradation and contamination were assessed by ethidium bromide staining of 28 s and 18 s ribosomal RNA on a 1% agarose gel [43]. The RNA integrity was then checked using an Agilent 2100 Bioanalyzer (Agilent Technologies, Palo Alto, CA, USA) with the cut-off value of RNA Integrity Number (RIN) ≥7. The RNA concentration was measured using a Qubit RNA HS Assay Kit in Qubit 2.0 Flurometer (Life Technologies, Carlsbad, CA, USA). The RNA-seq libraries were constructed using the TruSeq RNA Sample Prep Kit (Illumina, San Diego, CA, USA) following the manufacturer’s instructions. Briefly, mRNA was enriched using magnetic beads with Oligo (dT) and fragmented using divalent cations at elevated temperature. The RNA fragments were reverse transcribed into first strand cDNA with a six-base random primer, followed by second-strand cDNA synthesis, 3′ end repair and ligation of adapters. The ligated fragments were enriched by PCR to generate the final cDNA library. Finally, nine libraries (3 replicates for each kind of skin tissue) were constructed and sequenced on an Illumina HiSeq X Ten sequencer to generate 150 bp pair-end (PE) reads.

### De novo assembly and annotation

The raw paired-end reads were filtered to obtain high-quality clean reads using fastp 0.18.0 with the following parameters: -q 28 -u 20 -l 50–3 -W 4 -M 30 [44]. Owing to the absence of a reference genome of *G. eckloni*, the transcriptome was de novo assembled using Trinity 2.8.4 software based on merged clean paired-end reads [25]. The assembled unigenes were obtained after a clustering with CD-HIT-EST (c = 0.95) [26]. The unigenes were annotated by searching against six databases with the latest releases includingNr, Nt, COG, Swissprot, GO and KEGG. Three softwares were used for functional annotation with the e-value of 1e-10, including BLAST+ for Nt [45], Diamond for Nr, KOG, Swissprot and KEGG [46, 47], and Blast2GO for GO [48].

### Analysis of differentially expressed unigenes

Clean reads of each RNA-seq library were aligned to the assembled reference transcriptome to obtain unique mapped reads by using bowtie2 software [49]. The expression level of each unigene for each sample was calculated and normalized into FPKM (reads per kilobase of a gene per million reads) values by RSEM software [50]. All samples were divided into three groups, including SSS (shoulder scaled skin tissues), ASS (anal scaled skin tissues) and NSS (scaleless skin tissues). The count data were used to identify the differentially expressed unigenes between the group SSS and NSS, and ASS and NSS using the R package edgeR [51]. To limit false positive findings, unigenes with |log_2_FC| ≥ 2 and *p*-value ≤0.001 were identified as DEUs.

### GO and KEGG enrichment analysis of DEUs

To further understand the DEUs’ biological functions, GO and KEGG enrichment analysis were performed using the hypergeometric Fisher exact test in an online tool (OmicShare, www.omicshare.com/tools). The whole transcriptome was set as the background. The enriched GO and KEGG terms were confirmed with a threshold of false discovery rate (FDR)-corrected *p*-value < 0.05.

### qRT-PCR validation of DEUs

Ten DEUs were randomly selected for validation using qRT-PCR. The RNA samples used for RNA-seq library construction were also used for qRT-PCR. The first-strand cDNA was synthesized from 1 μg RNA using M-MLV Reverse Transcriptase (TaKaRa, Japan) with oligo (dT) primer. The qRT-PCR reactions were carried out on a Bio-Rad CFX-96 real-time PCR system (Bio-Rad, Hercules, USA) with the SYBR Premix ExTaq™ Kit (Takara, Japan). The relative expression levels were normalized to the quantification of β-actin using the 2^− ΔΔCT^ method [52].

## Supplementary Information

**Additional file 1: Table S1.** Summary of the sequencing data per sample in this study.

**Additional file 2: Figure S1.** Gene Ontology (GO) classification of all unigenes.

**Additional file 3: Figure S2.** KEGG classification of all unigenes.

**Additional file 4: Table S2.** Annotation of differentially expressed unigenes (DEUs) between NSS and others.

## Data Availability

All raw transcriptome data reported in this article have been deposited in the Genome Sequence Archive (GSA; http://gsa.big.ac.cn/) under accession number PRJCA003014.

## Abbreviations

DEUs: Differentially expressed unigenes
Eda: Ectodysplasin-A
EDAR: Ectodysplasin-A receptor
GO: Gene ontology
KEGG: Kyoto encyclopedia of genes and genomes
COGs: Cluster of orthologous groups of proteins
NGS: Next-generation sequencing
qRT-PCR: Reverse transcription quantitative PCR
QTP: Qinghai Tibetan Plateau
RNA-seq: RNA sequencing
SCCPs: Secretory calcium-binding phosphoproteins
Shh: Sonic hedgehog
TF: Transcription factors
