# Supplementary material for: Comparative transcriptome analysis of scaled and scaleless skins in Gymnocypris eckloni provides insights into the molecular mechanism of scale degeneration
Source: BMC Genomics. 2020 Nov 27;21:835. doi: 10.1186/s12864-020-07247-w (PMC7694923; doi:10.1186/s12864-020-07247-w)
Supplement: Supplementary file 1 — Additional file 1: Table S1. Summary of the sequencing data per sample in this study. [file 12864_2020_7247_MOESM1_ESM.doc]

**Supplementary Table S1.** Summary of the sequencing data per sample in this study

| **Samples** | **Raw reads** | **Clean reads** | **Q30** | **Q20** |
| --- | --- | --- | --- | --- |
| SSS1 | 47,797,046 | 46,573,512 | 94.13% | 98.13% |
| SSS2 | 54,367,792 | 52,987,940 | 93.58% | 97.90% |
| SSS3 | 62,424,338 | 60,605,790 | 93.05% | 97.67% |
| ASS1 | 44,906,926 | 43,603,042 | 93.88% | 98.02% |
| ASS2 | 60,955,522 | 59,621,420 | 94.89% | 98.44% |
| ASS3 | 45,215,412 | 43,986,648 | 92.85% | 97.60% |
| NSS1 | 66,872,900 | 65,299,628 | 93.97% | 98.05% |
| NSS2 | 68,763,990 | 67,390,338 | 94.63% | 98.34% |
| NSS3 | 54,689,322 | 53,401,718 | 94.34% | 98.21% |
