# Supplementary figures and images for: Comparative transcriptome analysis of scaled and scaleless skins in Gymnocypris eckloni provides insights into the molecular mechanism of scale degeneration
Source: BMC Genomics. 2020 Nov 27;21:835. doi: 10.1186/s12864-020-07247-w (PMC7694923; doi:10.1186/s12864-020-07247-w)

Additional file 2: Figure S1. Gene Ontology (GO) classification of all unigenes.

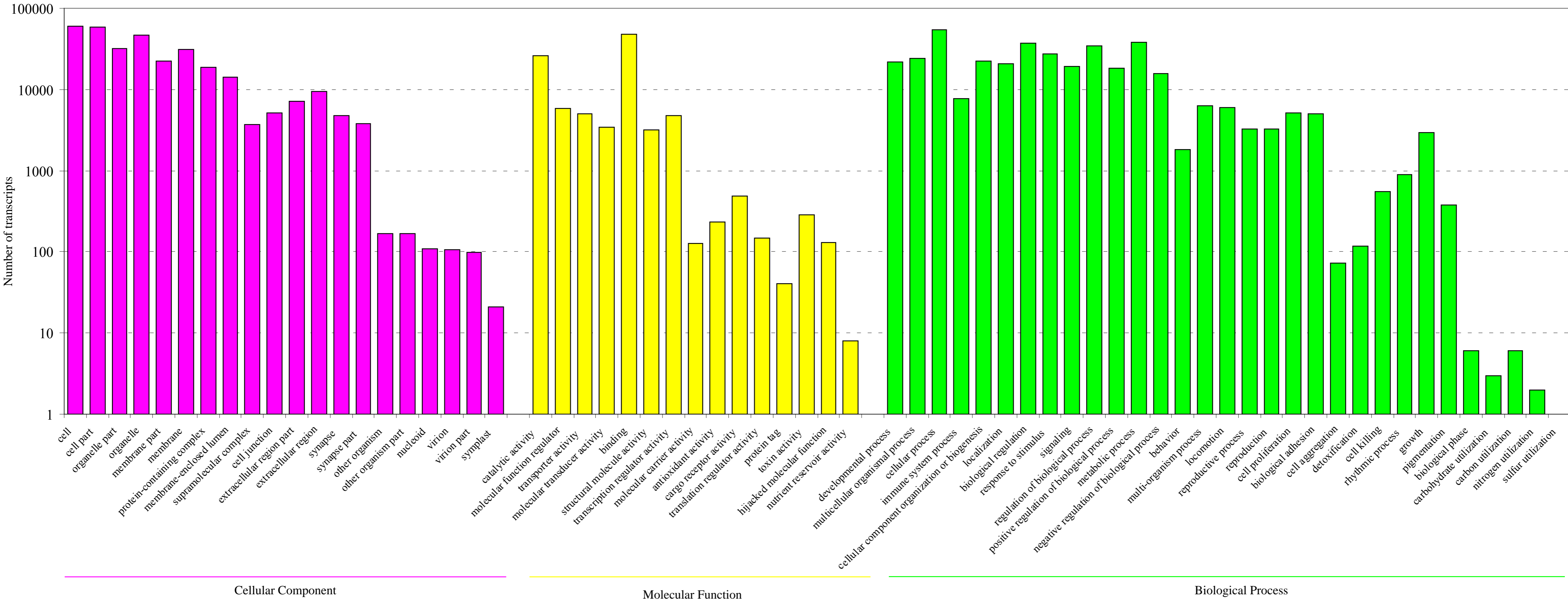

Supplement: Supplementary file 2 — Additional file 2: Figure S1. Gene Ontology (GO) classification of all unigenes. [file 12864_2020_7247_MOESM2_ESM.pdf]

## KEGG pathway annotation

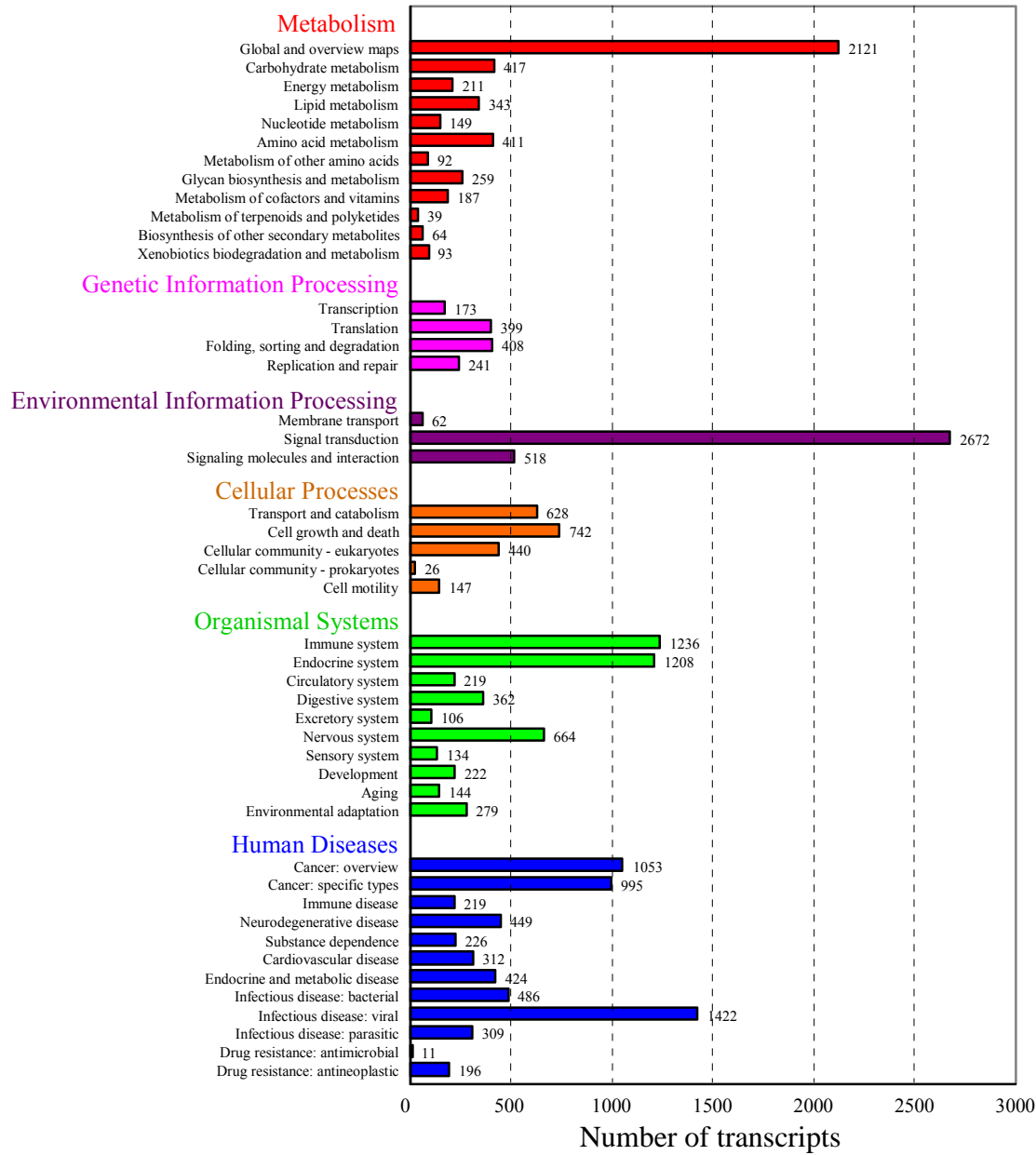

Supplement: Supplementary file 3 — Additional file 3: Figure S2. KEGG classification of all unigenes. [file 12864_2020_7247_MOESM3_ESM.pdf]
